# Molecular determinants of Guanylate Cyclase Activating Protein subcellular distribution in photoreceptor cells of the retina

**DOI:** 10.1038/s41598-018-20893-1

**Published:** 2018-02-13

**Authors:** Santiago López-Begines, Anna Plana-Bonamaisó, Ana Méndez

**Affiliations:** 1 Bellvitge Biomedical Research Institute (IDIBELL), Barcelona, Spain; 2 Department of Physiology, University of Barcelona School of Medicine-Bellvitge Health Science Campus, Barcelona, Spain

## Abstract

Retinal guanylate cyclase (RetGC) and guanylate cyclase activating proteins (GCAPs) play an important role during the light response in photoreceptor cells. Mutations in these proteins are linked to distinct forms of blindness. RetGC and GCAPs exert their role at the ciliary outer segment where phototransduction takes place. We investigated the mechanisms governing GCAP1 and GCAP2 distribution to rod outer segments by expressing selected GCAP1 and GCAP2 mutants as transient transgenes in the rods of GCAP1/2 double knockout mice. We show that precluding GCAP1 direct binding to RetGC (K23D/GCAP1) prevented its distribution to rod outer segments, while preventing GCAP1 activation of RetGC post-binding (W94A/GCAP1) did not. We infer that GCAP1 translocation to the outer segment strongly depends on GCAP1 binding affinity for RetGC, which points to GCAP1 requirement to bind to RetGC to be transported. We gain further insight into the distinctive regulatory steps of GCAP2 distribution, by showing that a phosphomimic at position 201 is sufficient to retain GCAP2 at proximal compartments; and that the bovine equivalent to blindness-causative mutation G157R/GCAP2 results in enhanced phosphorylation *in vitro* and significant retention at the inner segment *in vivo*, as likely contributing factors to the pathophysiology.

## Introduction

Retinal guanylate cyclases (RetGCs) and guanylate cyclase activating proteins (GCAPs) play a central role in photoreceptor cells of the retina. Together they are responsible for cGMP synthesis, the second messenger in the light response. When light is absorbed by the visual pigment, it triggers an enzymatic cascade that causes the hydrolysis of cGMP and a reduction of free cGMP levels. As a result, cGMP-gated channels in the plasma membrane close, causing a reduction of Na^+^ and Ca^2+^ influx and the hyperpolarization of the cell^1^. Once photoreceptors have responded to light, they must restore their cGMP to the dark levels to recover their sensitivity to light. This is achieved by a boost of cGMP synthesis caused by GCAPs activation of RetGC as the Ca^2+^ drops during the light response. This Ca^2+^-feedback signal to cGMP synthesis mediated by GCAPs plays an important role in termination of the light response and light adaptation^2,3^.

Mammalian rod and cone cells harbor two RetGCs, RetGC1 and RetGC2^4–7^. RetGC1 is more abundant than RetGC2 in photoreceptors, and is the isoform linked to inherited retinal dystrophies^8–11^. Two GCAP isoforms predominate in high mammals: GCAP1 and GCAP2. GCAP1 is present in rods and cones and primarily regulates RetGC1, while GCAP2 localizes mainly in rods, regulating both RetGC1 and RetGC2^10^. Numerous biochemical studies have characterized the mode of regulation of RetGC activity by GCAPs^10,12–17^. Basically, a homodimer of GCAPs is bound to a homodimer of RetGCs, with GCAPs being in a Ca^2+^-bound “inhibitor” conformation in the dark-adapted state; and switching to a Ca^2+^-free, Mg^2+^-bound, “activator” conformation as the Ca^2+^ drops in response to light.

RetGC1 and GCAPs have been linked to different forms of inherited retinal blindness^8,9,11^. To date, fifteen different mutations in GUCA1A encoding GCAP1 have been linked to autosomal dominant cone dystrophies^18–28^. Most of these mutations have the effect of decreasing GCAP1 Ca^2+^-binding affinity, resulting in constitutive activation of the cyclase independently of the light conditions, as shown by *in vitro* and *in vivo* studies^29–33^. One exception is the P50L GCAP1 mutation, that did not show an altered Ca^2+^ sensitivity of cyclase regulation *in vitro*^19^, and that might result toxic by other mechanisms *in vivo*. One mutation has been described in the GUCA1B gene encoding GCAP2, that has been linked to autosomal dominant retinitis pigmentosa: hG157R^34^. The effect of this mutation on the Ca^2+^-sensitivity of cyclase activation or the basis of its pathophysiology are unknown.

Little is known about the mechanisms that regulate RetGC/GCAPs protein assembly and transport to the outer segment in the context of living cells. RetGC1, an integral membrane protein, is synthesized at the cell soma. Its distribution to the outer segment compartment is predicted to rely on vesicular polarized trafficking. However, the molecular mechanisms underlying this process have remained elusive^35,36^. The soluble proteins GCAP1 and GCAP2 localize to the outer segment compartment, but also to the inner segment, perinuclear region and synaptic terminals of rods and cones^37–39^. Two independent lines of studies point to a regulated subcellular distribution of GCAPs. First, GCAP1 and GCAP2 fail to distribute to the outer segment compartment of rods and cones in the absence of functional RetGCs^35,36^, or when the stability of RetGCs is compromised^40^, which indicates that GCAPs distribution to rod outer segments requires RetGC. Second, GCAP2 subcellular distribution has been shown to be regulated by phosphorylation at Ser201 and 14-3-3 binding, depending on GCAP2 conformational state^41^.

While GCAPs dependence on RetGC for their distribution to the outer segment has been established, it is not known whether it involves a direct interaction. Alternatively, GCAPs could simply incorporate by default to vesicle transport carriers guided by RetGC, as proposed for PDE6 in rods, and PDE alpha and transducin in cones^35,36^. In addition, GCAP1 and GCAP2 are myristoylated at Gly2 at the NH2-terminus. Acylation has been shown to be critical for the subcellular distribution of numerous membrane-associated proteins in photoreceptor cells^42–44^. However, whether myristoylation affects GCAPs subcellular distribution in living photoreceptors has not been directly addressed.

Recent structural-functional studies have provided a precise mapping of the residues in GCAP1 involved in primary binding to the cyclase^45–48^. Among the surface exposed residues at the binding interface encompassing EF-hand 1, Lys23 has been shown to be essential for GCAP1 binding to RetGC^45,46^. Mutation of Lys23 to aspartic acid results in >90% loss of GCAP1 capacity to activate the cyclase and the inability of GCAP1 to colocalize with RetGC1 in co-transfected HEK293 cells^46^. Other GCAP1 residues (Met26, Lys85, Trp94) have been shown to contribute strongly to RetGC activation, without being critical for GCAP1 primary binding to the cyclase^46^. Mutations at these positions block GCAP1 cyclase activation, but do not preclude GCAP1 co-localization with the cyclase in cotrasfected cells^46^.

Based on this information, we here addressed whether GCAP1 direct binding to RetGC1 is required for GCAP1 distribution to the rod outer segment compartment and whether this distribution is affected by GCAP1 myristoylation. We expressed a GCAP1 mutant impaired at RetGC1 binding (K23D/GCAP1), and one that preserved primary binding but failed to activate RetGC1 (W94A/GCAP1) as transient transgenes in the rods of GCAP1/2 double knockout mice. We show that precluding GCAP1 binding to RetGC1, but not cyclase activation per se, prevented its distribution to rod outer segments. Preventing GCAPs myristoylation affected GCAP1 distribution more substantially than GCAP2, consistent with the reported effect of GCAP1 myristoylation on GCAP1 apparent affinity for the cyclase *in vitro*^49,50^. Taken together our results show that a decrease in GCAP1 binding affinity for RetGC severely impairs GCAP1 translocation to the outer segment.

In addition, we further confirm that GCAP2 phosphorylation at Ser201 and 14-3-3 binding retain GCAP2 at the inner segment^41^. Originally shown on a transgenic line expressing a GCAP2 mutant locked in its Ca^2+^-free conformation (EF^−^GCAP2), we here show that a negatively charged residue at position 201 of wildtype GCAP2 retains the protein at the inner segment. Furthermore, as we had previously predicted^41^, we show that bG161R/GCAP2 (bovine equivalent to disease-linked human mutation G157R/GCAP2) is significantly retained at the inner segment in a high fraction of photoreceptor cells, consistent with an observed higher susceptibility of the mutant to phosphorylation *in vitro*. We propose that an accumulation of GCAP2 at the inner segment likely contributes to G157R/GCAP2 pathophysiology in adRP.

## Results

### Molecular determinants of GCAP1 subcellular distribution *in vivo*

To study whether GCAP1 requires direct binding to RetGC to be distributed to the outer segment compartment, we expressed wiltype GCAP1 and a GCAP1 mutant impaired at RetGC1 binding (K23D) as transient transgenes in the rods of GCAP1/2 double knockout mice. Lys23 has been shown to be an essential residue in the EF-hand 1 region of GCAP1 that constitutes the binding interface with the cyclase^45,46^.

We attained transient transgenesis in rods by expressing the human GCAP1 cDNA and corresponding mutants with the mouse opsin promoter (4.4-kb version). Expression vectors were transfected in the retina of newborn mice by *in vivo* DNA electroporation after subretinal injection, and retinas were processed at p25-p30 for indirect immunofluorescence analysis of GCAP1 (see Methods). Recombinant DNA transfection by electroporation resulted in the mosaic expression that characterizes this technique^51–54^. A somewhat lower transfection efficiency was obtained in this study compared to other studies^51–54^, likely due to the use of the 4.4 kb rather than the 2.2 kb version of the mouse opsin promoter which yields larger DNA vectors.

**Figure 1:**
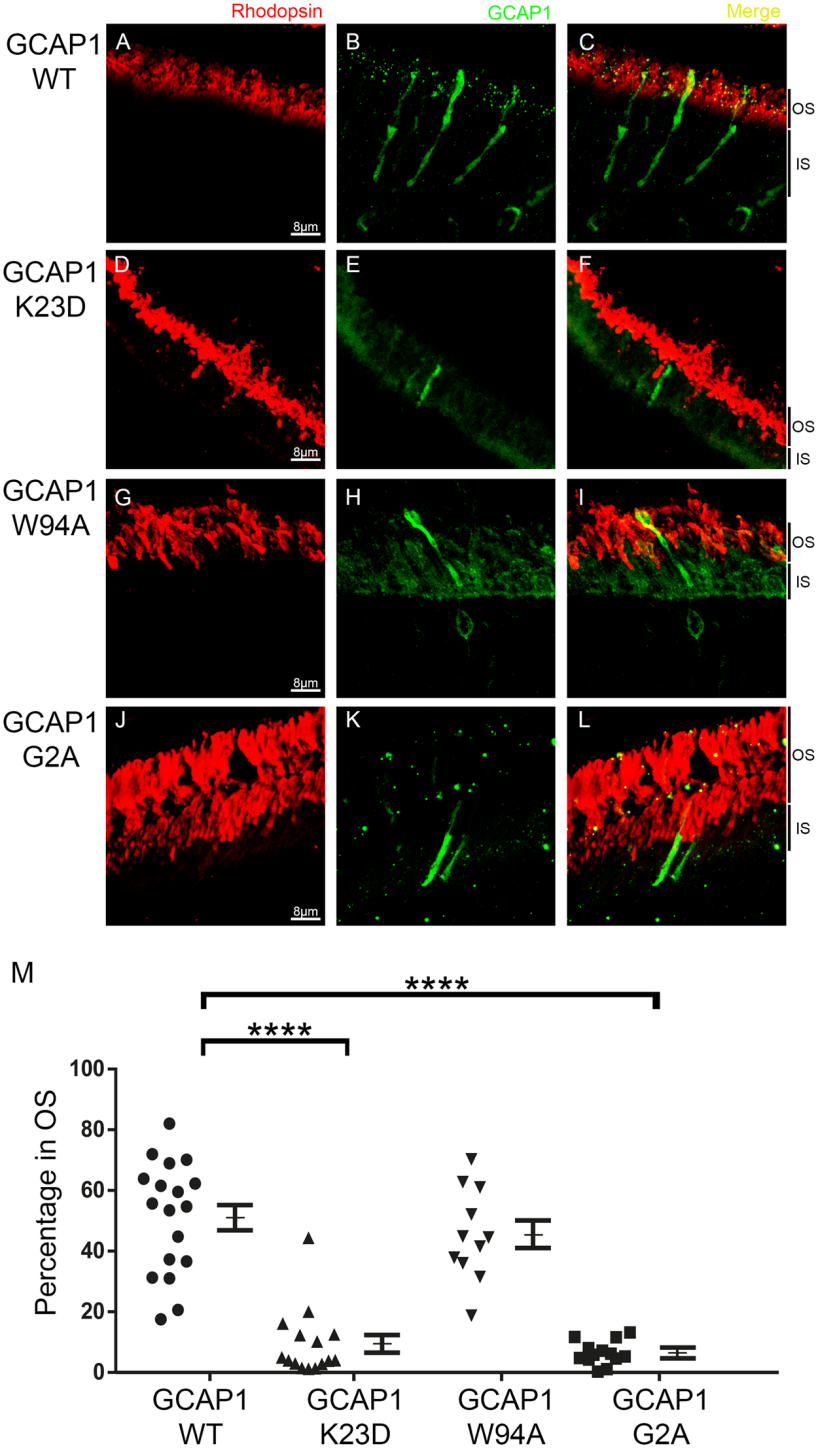
Molecular determinants of GCAP1 distribution to rod outer segments *in vivo*. Wildtype and different mutant forms of GCAP1 were expressed as transient transgenes in the rods of GCAP1/GCAP2 knockout mice. Mosaicism results from the *in vivo* DNA electroporation method of transfection. GCAP1 WT (green signal in **B**,**C**) distributed 50:50% between the inner and outer segment compartments. K23D/GCAP1 (green signal, **E**,**F**) was retained at the inner segment, as if its distribution to rod outer segments was precluded. W94A/GCAP1 (green signal, **H**,**I**) reproduced the wildtype localization. G2A/GCAP1 (green signal, **K**,**L**) was retained at the inner segment. In red, rhodopsin mAb1D4 labels the rod outer segment layer (**A**,**D**,**G**,**J** and merged images). M. Percentage of GCAP1 signal at the outer segment compartment (from the combined signal at outer and inner segments). Mean values are indicated, with bars representing the standard error. Mean ± SEM were: WT (●) 51.30 ± 4.38, n = 18; K23D (▲) 9.48 ± 2.92, n = 15; W94A (▼) 45.58 ± 4.55, n = 11; G2A (■) 6.49 ± 3.73, n = 13. T-test was used to determine statistical significance versus WT. For G2A and K23D mutants, p-value < 0.0001. At least three injected animals per construct were analyzed, showing consistent results. The outer segment length may vary depending on the position of the acquired field-image on the retina section.

The distribution of the transfected wildtype GCAP1 protein between the inner and outer segments of GCAP1/2 dKO rods was determined in 18 individual cells, taken from 7 fields from 5 mice (images provided in Figure S1). A representative image is shown in Fig. 1, panels A-C. The GCAP1 signal at the outer segment was determined in each cell, and expressed as a fraction of the total GCAP1 signal at both compartments (plotted in Fig. 1M). The percentage of GCAP1 at the outer segment was determined to be 51.30 ± 4.38 (n = 18). This intracellular GCAP1 distribution reproduced that of endogenous GCAP1 in murine rods^39^.

In contrast, the K23D/GCAP1 mutant, impaired at binding RetGC, was retained at the inner segment and failed to be transported to rod outer segments. A representative example is shown in Fig. 1D–F. A total of 15 individual cells were analyzed from 11 fields from 4 mice (images provided in Fig. S2), resulting in a mean percentage of GCAP1 distribution to rod outer segments of 9.48 ± 2.92 (Fig. 1M). This result indicated that GCAP1 depends on its binding to RetGC1 for its distribution to rod outer segments, while it does not distinguish whether the requirement relies on primary binding or subsequent cyclase activation.

To prove that the requirement for GCAP1 translocation relied on GCAP1 binding affinity to RetGC, and not on GCAP1 cyclase activating capacity, we carried the W94A/GCAP1 mutant.

Trp94 is one of a few GCAP1 residues (Met26, Lys85, Trp94) that contribute strongly to RetGC activation but are not critical for GCAP1 primary binding to the cyclase^45,46^. Mutations at these positions block GCAP1 cyclase activation, but do not preclude the co-localization of GCAP1 with the cyclase in cotrasfected cells^46^. We here show that the W94A/GCAP1 mutant presented a similar distribution to the wildtype protein, resulting in a percentage of 45.58 ± 4.55 (n = 11) of protein distributed to rod outer segments (representative image shown in Fig. 1G–I; distribution of the cell population shown in Fig. 1M). The 11 cells analyzed are shown in Fig. S3, and they come from 9 fields from 3 mice. These results showed that the loss of RetGC activation capacity by itself did not significantly affect translocation.

Taken together our results show that the loss of GCAP1 activating capacity of RetGC per se (W94A/GCAP1) was not sufficient to cause retention, while a pronounced decrease in GCAP1 binding affinity for RetGC (K23D/GCAP1) severely impaired translocation.

In photoreceptor cells, several acylated proteins involved in phototransduction have been shown to interact with lipid-binding proteins that influence or are critical for their subcellular distribution^42–44^. To assess whether the myristoyl group at GCAP1 Gly2 at the NH2-terminus is required for GCAP1 distribution to rod outer segments *in vivo*, we expressed the mutant G2A/GCAP1. This mutant was massively retained at the inner segments (representative example shown in Fig. 1J–L). This nearly complete retention was consistently observed in 13 individual cells analyzed from 9 fields from 3 mice (images provided in Fig. S4), resulting in a percentage of GCAP1 distribution to rods of 6.49 ± 3.73 (Fig. 1M). This result indicated that myristoylation of GCAP1 is required for its proper distribution to the outer segments *in vivo*.

Previous *in vitro* studies have shown that GCAP1 myristoylation increases the apparent binding affinity of GCAP1 to RetGC^49,50^. Therefore our results indicate again that a decrease in GCAP1 affinity for RetGC hinders GCAP1 translocation.

Taken together our results show that GCAP1 transport to rod outer segments strongly depends on GCAP1 binding affinity to RetGC1.

### Molecular determinants of GCAP2 subcellular localization in photoreceptor cells

Whether GCAP2 binding to RetGCs is a prerequisite for its trafficking to rod outer segments could not be tested because the target binding domain in GCAP2 has not been mapped as precisely^55^. However, we have previously reported that GCAP2 subcellular distribution presents additional regulatory steps, not reported in GCAP1. In wildtype mice raised under standard 12 h dark:12 h light cycles, about 50% of GCAP2 is phosphorylated at Ser201^41^. *In vitro*, it is the Ca^2+^-free form of GCAP2 that is phosphorylated more efficiently^41,56^. Our previous study showed that *in vivo*, a mutant form of GCAP2 locked in its Ca^2+^-free form (EF^−^GCAP2) was hyperphosphorylated and retained at the inner segment by 14-3-3 binding^41^, conceivably due to 14-3-3 interfering with GCAP2 binding to RetGC. What our previous study did not directly prove is that phosphorylation of the wildtype protein would cause its retention at the inner segment compartment as well.

We here transfected rods with the phosphorylation mimic mutant S201D/GCAP2, to test whether introducing a negative charge at position 201 of the wildtype protein is sufficient to preclude its distribution to rod outer segments. As a control, we also transfected mice with S201G/GCAP2.

**Figure 2:**
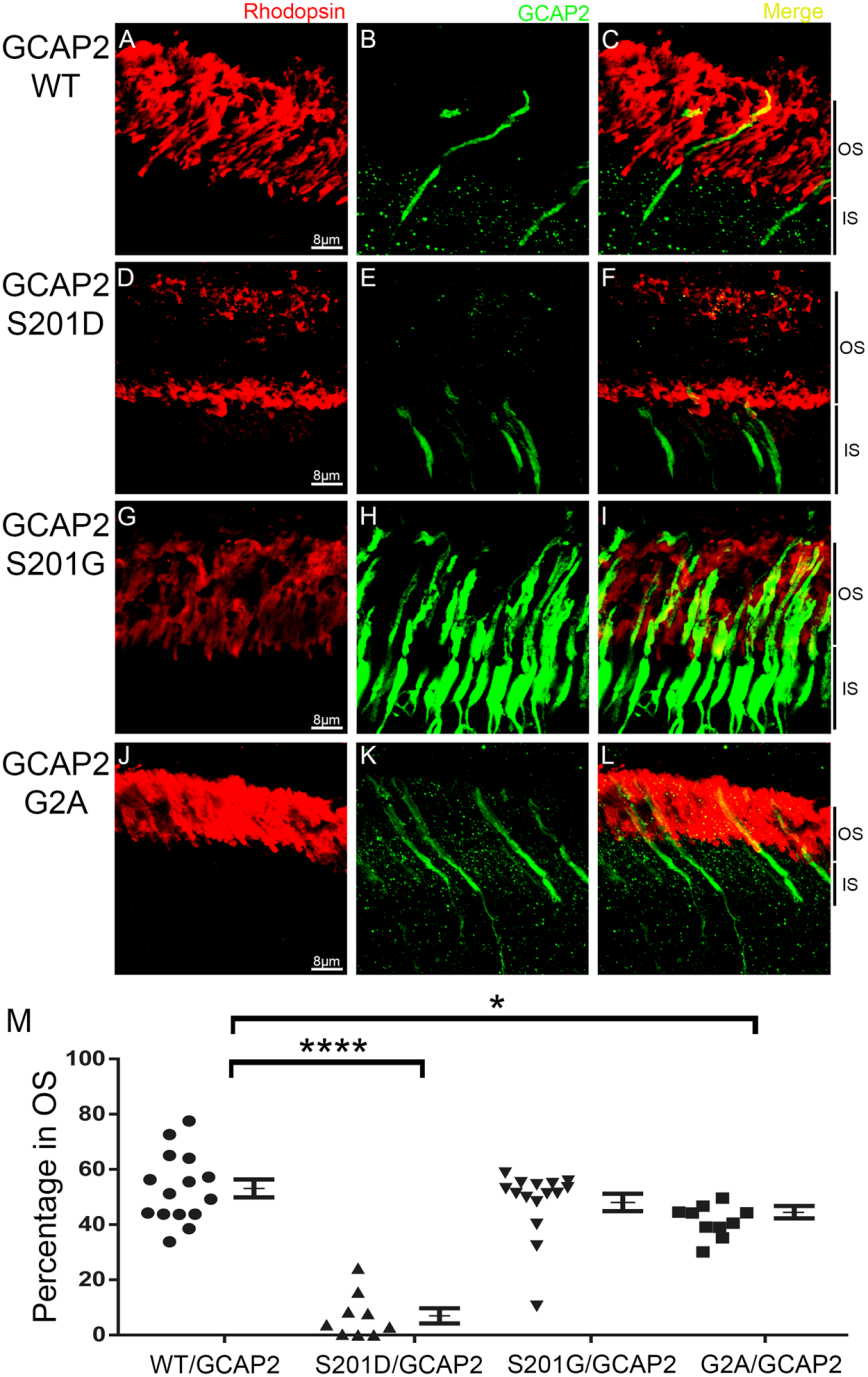
Molecular determinants of GCAP2 distribution between rod inner and outer segments *in vivo*. Wildtype and mutant GCAP2 constructs were used to transfect the rods of GCAP1/2 knockout mice. GCAP2 WT (**B**,**C**) distributed between the inner and outer segment compartments to about 50%:50%. S201D-GCAP2, a constitutive mimic of phosphorylated GCAP2 was mainly retained at the inner segments (**D**–**F**), while S201G-GCAP2 (**G**–**I**) reproduced the wildtype localization. S201G/GCAP2 mutant yielded a much more intense signal than the wildtype electroporated protein using similar image acquisition parameters. G2A/GCAP2 (**K**,**L**) distribution to rod outer segments diminished by 12% (P-value < 0.01), but was clearly not impaired. In red, rhodopsin mAb 1D4 labels the rod outer segment layer (**A**,**D**,**G**,**J** and merged images). M. Percentage of signal at rod outer segments (expressed as a fraction of the combined signal in outer and inner segments). Mean values are indicated. Values Mean ± SEM: WT (●) 53.11 ± 3.25, n = 15; S201D (▲) 6.99 ± 2.75, n = 9; S201G (▼) 48.01 ± 3.18, n = 15; G2A (■) 41.34 ± 1.83, n = 10. T-test was used to determine statistical significance versus WT. For G2A mutant, p-value < 0.01; for S201D mutant, p-value < 0.0001. Three injected animals were analyzed for the GCAP2 wildtype construct, while two injected animals were analyzed for S201D/GCAP2; S201G/GCAP2 and G2A/GCAP2; showing consistent results. The outer segment length may vary depending on the position of the acquired field-image on the retina section.

When wildtype GCAP2 was transfected into GCAP1/GCAP2 knockout rods, it distributed to about 50:50% between rod inner and outer segments [53.11% ± 3.25 in 15 individual cells]. A representative image is provided in Fig. 2A–C, and images of 14 additional cells acquired from another 11 fields from 3 mice are provided in Fig. S5. This distribution reproduced the endogenous localization of GCAP2 in wildtype mice^41^. The constitutive mimic of phosphorylated GCAP2, S201D/GCAP2, was retained at the inner segment compartment (representative image in Fig. 2D–F; and images of 8 cells in 4 fields from at least 2 mice shown in Fig. S6). The number of cells analyzed for this construct was limited despite the fact that we injected many animals. We observed that this construct resulted in changes in cell morphology when expressed at relatively high levels, possibly due to toxicity, as shown in Fig S6. Many of the injected mice did not show transfected cells for this particular construct, possibly because expression of this mutant causes cell death. In contrast, S201G/GCAP2 distributed to the outer segment normally in most cells analyzed (Fig. 2G–I). Fourteen cells analyzed from 4 fields of two mice are also shown in Fig. S6. Interestingly, the GCAP2 signal appeared reduced in rods transfected with S201D/GCAP2, and enhanced in rods transfected with S201G/GCAP2, which may be indicative of a role of phosphorylation at Ser201 in signaling protein degradation (see Discussion).

In order to test whether myristoylation of GCAP2 affected its subcellular distribution, we transfected rods with G2A/GCAP2. Abolishing myristoylation of GCAP2 did not have the severe effect observed for GCAP1, but it diminished GCAP2 distribution to rod outer segments by 12% (Fig. 2J–L). Figure S6 shows the 10 cells analyzed, distributed in at least three fields from two mice. Mean value was 41,34 ± 1,83. Therefore, our results point to a facilitation of GCAP2 localization to rod outer segments by the myristoyl group, even if not strictly required.

Taken together, our results are consistent with the previously proposed model of GCAP2 subcellular localization^41^, and also point to the myristoyl group at facilitating translocation to the outer segment compartment.

### Subcellular distribution of P50L/GCAP1 CORD-associated mutant

**Figure 3:**
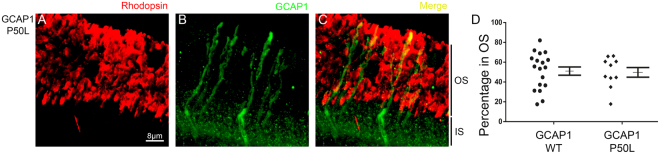
Subcellular distribution of P50L-GCAP1. Transient expression of P50L/GCAP1 in the rods of GCAP1/2 knockout mice shows a subcellular distribution of the mutant (green signal in **B**,**C**) similar to the wildtype protein. In red, rhodopsin mAb 1D4 labels the rod outer segment layer (**A**,**C**). (**D**) Percentage of GCAP1 signal at rod outer segments (expressed as a fraction of the combined signal in outer and inner segments). Mean ± SEM. WT (■) 51.30 ± 4.38, n = 18; P50L (♦) 49.73 ± 4.88, n = 10. The population of wildtype GCAP1 cells is that from Fig. 1, and is shown here for a direct illustrative comparison with the P50L-cell population. Two injected mice were analyzed for this construct, showing consistent results.

Fifteen mutations in GUCA1A encoding GCAP1 have been linked to autosomal dominant cone rod dystrophy (adCORD) or macular dystrophies^18–28^. Most mutations decrease the Ca^2+^ binding affinity of the protein, altering the Ca^2+^ sensitivity of cyclase activation in a manner that results in constitutive activity^29–33^. However, the cone dystrophy-associated mutation P50L appears to be an exception. This mutant was reported to have a similar Ca^2+^-sensitivity as wildtype GCAP1 in guanylate cyclase activity assays, but to have a reduction in thermal stability^19^. Because of the lack of an alteration in Ca^2+^-sensitivity regulation of the cyclase that could explain its pathophysiology, we set to examine the effect of this mutation on the intracellular distribution of GCAP1. P50L/GCAP1 was transiently expressed as a transgene in the rods of GCAP1/2 knockout mice. The analysis of these mice showed that the P50L mutation did not affect GCAP1 subcellular distribution. P50L/GCAP1 showed a subcellular localization similar to the wildtype protein (Figs 3 and S7).

### An autosomal dominant Retinitis Pigmentosa mutation in GUCA1B encoding GCAP2 affects its subcellular distribution

**Figure 4:**
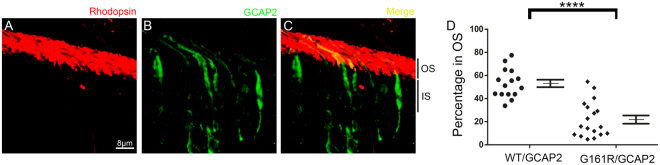
Subcellular distribution of bG161R/GCAP2 in transfected rods of GCAP1/2 knockout mice. The bG161R/GCAP2 mutant showed a substantially impaired distribution to rod outer segments in the majority of transfected cells (green signal in **B**,**C**), and population analysis in (**D**). In red, rhodopsin 1D4 mAB stains the rod outer segment layer. In 11 out of 18 cells, bG161R/GCAP2 distribution to rod outer segments was <20%. Only in 3 out of 18 analyzed cells the extent of mutant distribution was similar to the wildtype protein. In the graph in (**D**), the population of cells from Fig. 2 is represented next to the population of bG161R/GCAP2 cells, for a direct illustrative comparison of both distributions. G161R/GCAP2 Mean ± SEM: 21.93 ± 3.64 (n = 18) [Versus wildtype GCAP2, 53.11 ± 3.25, n = 15]. T-test, p-value < 0.0001. Three injected animals were analyzed for this construct, showing similar results.

We have previously shown that GCAP2 retention at the inner segment locked in its Ca^2+^-free unstable form is associated with cell death and severe retinal degeneration^41^. Therefore, we here addressed whether the human mutation associated to autosomal dominant retinitis pigmentosa (hG157R)^34^ affected GCAP2 sensitivity to Ca^2+^ and consequently GCAP2 subcellular distribution. We expressed the G157R equivalent mutation in the bovine GCAP2 isoform, bG161R/GCAP2, as a transient transgene in mouse rods of the GCAP1/2 knockout. The subcellular localization of bG161R/GCAP2 was analyzed in 18 independent cells from 8 fields from three mice (Fig. S8). A representative image is shown in Fig. 4. The analysis revealed a rather widespread distribution in the population of transfected cells, but it was clear that more than 50% of transfected cells (10 out of 18) showed < 20% GCAP2 distribution to rod outer segments (Fig. 4).

**Figure 5:**
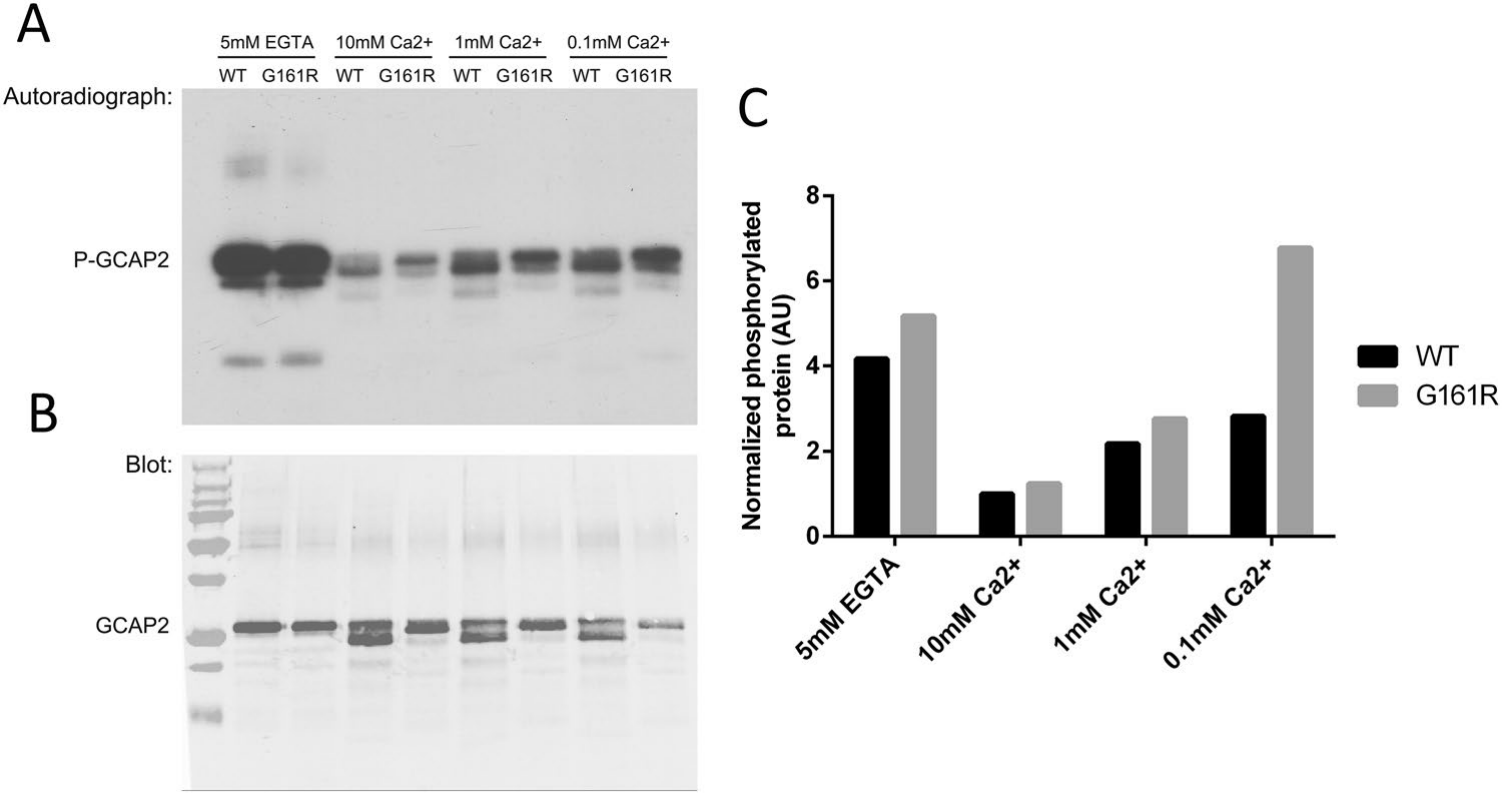
*In vitro* phosphorylation of recombinant bGCAP2 and bG161R/GCAP2 by PKG. 20 μg of bGCAP2 or bG161R/GCAP2 were incubated for 10 min in 40 μl of buffer with fixed EGTA or CaCl_2_ concentrations: 5 mM EGTA; 10 mM CaCl_2_; 1 mM CaCl_2_; or 0.1 mM CaCl_2_. *In vitro* phosphorylation reactions were performed by adding 10x phosphorylation buffer, 3 µCi of ^32^P-γATP and 5 units of PKGIα to a final volume of 50 μl, and incubating for 30 min at room temperature. Samples were resolved in a 15% SDS-PAGE and transferred to a nitrocellulose membrane. (**A**) Autoradiograph of the membrane, showing the phosphorylation level of GCAP2 and G161R/GCAP2. Phosphorylation of both proteins was maximal at 5 mM EGTA, when both proteins are at their Ca^2+^-free conformation. Minimal phosphorylation was observed at 10 mM CaCl_2_. With decreasing CaCl_2_, a progressively higher extent of phosphorylation was observed at bG161R/GCAP2, particularly noticeable at 0.1 mM CaCl_2,_ a concentration within the physiological range of free [Ca^2+^]_i_ in photoreceptors. Wildtype and bG161R/GCAP2 proteins showed different mobility patterns. Intriguingly, the autoradiograph revealed an “intermediate Ca^2+^-shift” in the wildtype protein not readily appreciated by Western; while the most abundant Ca^2+^-bound form of the protein, with a lower mobility, was only revealed by Western blot and was clearly not phosphorylated. (**B**) Immunoblot of previously exposed nitrocellulose membrane. Note that a careful superimposition of the Western and autoradiograph images reveals that the lower band in wildtype lanes detected with the Ab is at a lower position than ^32^P-labeled bands. bG161R/GCAP2 did not show the lower Ca^2+^-shift band observed in the wildtype protein, indicating that its Ca^2+^ sensitivity was altered by the mutation. First lane: molecular weight standards (Biorad #161-0373) with a distinctive brightness resulting from the color to greyscale conversion of an Odyssey image (protein stardards imaged in red while protein samples in green channel). (**C**) Histogram depicting the level of phosphorylation of wildtype and mutant proteins, normalized by the total amount of protein observed by western, expressed as a function of the wildtype protein at 10 mM CaCl_2_. bG161R/GCAP2 is substantially more phosphorylated than the wildtype at 0.1 mM CaCl_2_, a calcium concentration in the physiological range of intracelular [Ca^2+^] in photoreceptors.

Because the residue that is phosphorylated in GCAP2, Ser201, is preferentially exposed in the Ca^2+^-free form of GCAP2, the retention of bG161R/GCAP2 at the inner segment could be explained if the blindness mutation resulted in higher exposure of Ser201 at the physiological range of Ca^2+^ concentrations. That is, if G161R mutation decreased GCAP2 Ca^2+^ sensitivity so that the Ca^2+^-dependence of S201 phosphorylation was affected. To test this possibility, *in vitro* phosphorylation assays were performed with myristoylated bGCAP2 and bG161R/GCAP2 recombinant proteins, at a range of calcium concentrations (Fig. 5). *In vitro* phosphorylation was performed with cGMP dependent protein kinase (PKG). The kinase that phosphorylates GCAP2 *in vivo* has not been identified, but it is known that GCAP2 is a substrate of PKG *in vitro*^41,56^. Preceding the *in vitro* phosphorylation reactions, recombinant proteins were preincubated for 10 min in buffer with fixed EGTA/ Ca^2+^ concentrations: 5 mM EGTA; 10 mM CaCl_2_; 1 mM CaCl_2_ or 0.1 mM CaCl_2_. These EGTA or CaCl_2_ concentrations were maintained during the 30 min phosphorylation reactions in the presence of ^32^P-ATPγ (see Methods). Under these conditions, phosphorylation of both proteins was maximal in 5 mM EGTA conditions, and was minimal in 10 mM CaCl_2_ (Fig. 5A). However, at 1 mM CaCl_2_ and particularly at 0.1 mM CaCl_2_, the mutant protein was phosphorylated to a higher extent than the wildtype (Fig. 5A). This was more evident when the level of phosphorylation of wildtype and mutant proteins was determined from the autoradiograph and normalized by the total amount of protein observed by GCAP2 immunoblot of the membrane (Fig. 5A and B), and expressed as a function of the wildtype protein at 10 mM CaCl_2_ (histograms at Fig. 5C). It became clear that bG161R/GCAP2 was substantially more phosphorylated than the wildtype protein at 0.1 mM CaCl_2_, the calcium concentration that approximates the intracellular calcium concentration of photoreceptor cells in darkness^57^. Results shown are representative of two independent experiments.

Taken together our results point to an increased susceptibility of bG161R/GCAP2 to phosphorylation at Ser201 in photoreceptor cells independently of the light conditions (along the physiological range of intracellular calcium concentrations), that is consistent with the retention of the protein observed. Because we have previously shown that an accumulation of Ca^2+^-free GCAP2 at the inner segment results in photoreceptor cell death and severe retinal degeneration^41^, we infer that mutant GCAP2 retention at the inner segment will contribute to the pathophysiology of adRP hG157R/GCAP2.

## Discussion

In this study, we gain insight into the molecular determinants of subcellular localization of Guanylate Cyclase Activating Proteins GCAP1 and GCAP2 in photoreceptor cells. A recent structural-functional study has delineated the surface binding domain to RetGC1 in GCAP1. A detailed mapping of the surface exposed residues in GCAP1 by mutagenesis followed by co-localization and *in vitro* cyclase activity assays allowed the identification of residues involved in primary binding to the cyclase versus residues dispensable for primary binding but required for subsequent activation^45–48^. Based on this structural information, we here addressed whether GCAP1 direct binding to the cyclase is required for its distribution to rod outer segments *in vivo*.

We show that mutating Lys23, an amino acid required for primary binding and activation of RetGC^45–48^, severely impaired GCAP1 translocation to rod outer segments of transient transgenic mice. Mutating Trp94, dispensable for primary binding but required for post-binding activation^46^, did not noticeably affect GCAP1 translocation. Taken together our results showed that the loss of GCAP1 cyclase activation capacity by itself did not have a noticeable effect on GCAP1 translocation, while the loss of GCAP1 binding affinity for RetGC caused the retention of GCAP1 at the inner segments. Therefore our results indicate that GCAP1 translocation to the outer segment depends on GCAP1 binding affinity for RetGC, and point to the RetGC/GCAP complex being assembled previous to its transport. Our results are consistent with previous results showing that GCAP1 and GCAP2 fail to be transported to rod outer segments in the absence of a functional RetGC in the RetGC1/2 knockout mice or the rd3 mice^35,36,40^. Future work will be required to confirm this mechanistic insight into RetGC/GCAPs transport by using more powerful imaging techniques: e.g. fluorescence resonance energy transfer, or superresolution microscopy techniques.

GCAP1 and GCAP2 are both myristoylated at Gly2 at the NH2-terminus. The lipid moiety of many membrane-associated proteins involved in the light response has been involved in modulating protein solubility and subcellular distribution^42–44^. Therefore, we studied whether the myristoyl group was required for GCAP1 and GCAP2 distribution to rod outer segments *in viv*o. Precluding the myristoylation of GCAPs *in vivo* had a significant effect on their subcellular localization. Unmyristoylated GCAP1 was largely retained at the inner segment (only 6% of the protein distributed to the outer segments, Fig. 1). Unmyristoylated GCAP2 distributed to rod outer segments, although to a lesser extent than the myristoylated protein (12% less, Fig. 2). The effect of precluding myristoylation on GCAPs distribution *in vivo* was unanticipated. GCAP1 and GCAP2 do not exhibit the “Ca^2+^-myristoyl switch” reported for recoverin, in which the fatty acid is extruded from the hydrophobic pocket in response to Ca^2+^ binding, contributing to membrane attachment^58^. Crystallographic and NMR studies have documented that the myristoyl group in GCAP1 and GCAP2 is maintained inside the hydrophobic pocket both in the “inhibitory” and the “activator” states, not being exposed^45,50,55,59^. It has been proposed that the myristoyl group in GCAP1 contributes to connect Ca^2+^-binding affinity to the extent of cyclase activation by establishing a dynamic connection between Ca^2+^-binding at EF-4 and the RetGC recognition surface in GCAP1^50^. In RetGC activation assays, the apparent affinity of myristoylated GCAP1 for RetGC was determined to be 5- to 7-fold higher than that of unmyristoylated GCAP1^49,50^. This difference in GCAP1 apparent affinity for the cyclase might explain its retention at the inner segment when myristoylation is precluded, highlighting the physiological relevance of the myristoyl group *in vivo*.

It cannot be dismissed that a yet-to-be-identified acyl-binding protein could play a role in GCAP1 distribution to rod outer segments. An acyl-binding protein, UNC119, has been reported to bind to the myristoylated N-termini of G-protein α-subunits, being involved in transducin α redistribution between rod outer segment and proximal compartments during light- and dark-adaptation periods^42,44^. However, GCAP1 failed to interact with UNC119A^42^, and reports are mounting that place the GCAP1 myristoyl group buried inside the molecule in both conformational states^45,50,55,59,60^. Therefore this possibility seems unlikely.

Interestingly, there are common and distinctive aspects in the mechanisms that determine GCAP1 and GCAP2 subcellular distribution. Precluding myristoylation of GCAP2 had an statistically significant effect on subcellular distribution, although lesser than that on GCAP1. Biochemical studies have determined that myristoylation of GCAP2 has only a minor effect on Ca^2+^ binding affinity and the extent of cyclase activation, compared to GCAP1^15,49,50^. The myristoyl in GCAP2 has been proposed to serve to thermally stabilize the protein^61^, and this effect might contribute to explain the different extent of myristoylated and unmyristoylated GCAP2 distribution to rod outer segments.

A distinctive aspect of GCAP2 subcellular distribution is that it is regulated by phosphorylation. In wildtype mice reared in standard cyclic light, about 50% of GCAP2 is phosphorylated at Ser201, that sequesters the protein at the inner segment^41^. Phosphorylation at Ser201 does not affect RetGC activation *in vitro*^56^. Therefore, it is unlikely that phosphorylation alone interferes with GCAP2 binding to RetGC *in vitro* or *in vivo*. However, we have shown that phosphorylation at Ser201 triggers 14-3-3 binding to GCAP2 *in vivo*^41^, an step that could conceivably hinder GCAP2 binding to RetGC, in a similar way as 14-3-3 binding to phosphorylated phosducin masks its binding sites to Gβγ^62,63^.

In this study we further validate this regulatory mechanism of GCAP2 subcellular distribution, by showing that a constitutive mimic mutant of phosphorylated GCAP2 (S201D/GCAP2) is largely retained at the inner segment. We also show that precluding phosphorylation at this position (S201G/GCAP2) results in an inner: outer segment distribution of 50%: 50% similar to the wildtype protein. This is consistent with our previous interpretation that the maximal distribution to the rod outer segment of GCAP2 is about 50%^41^. Interestingly, the S201G/GCAP2 mutant presented a noticeably higher level of expression than the wildtype or S201D/GCAP2 proteins (Fig. 2). This result suggests that phosphorylation at Ser201 might be a signal that favors GCAP2 degradation. Future studies will be addressed at characterizing the turnover rate of these mutants in transfected cells.

A mutation in GUCA1B encoding GCAP2 has been linked to retinitis pigmentosa, hG157R^34^. This residue is localized in EF-hand 4, and is therefore expected to diminish GCAP2 Ca^2+^ binding affinity. A reduced Ca^2+^ sensitivity of guanylate cyclase regulation could result in constitutive cyclase activity. However, we have reported that when GCAP2 is Ca^2+^ insensitive it fails to be transported to rod outer segments, but leads to a severe retinal degeneration by a mechanism independent of cGMP. Toxicity in this case results from EF^−^GCAP2 accumulation at the inner segment^41^. We here show that the equivalent bovine mutation to hG157R (bG161R) causes the retention of 80% of GCAP2 at the inner segment in more than half of transfected photoreceptors (11 out of 18 cells, Fig. 4). In about a fifth of transfected rods bG161R/GCAP2 distributed normally. The dispersion of GCAP2 distribution of bG161R-GCAP2 transfected cells might result from variability of GCAP2 expression levels among cells. GCAP2 expression levels have been shown to vary significantly from cell to cell in previous genetic studies, both under the endogenous promoter^17^ and when expressed in stable transgenic mice under the rod opsin promoter used in this study^2^. Ultimately, the bG161R mutation led to GCAP2 retention at the inner segment in a high fraction of cells. The bG161R mutation also altered the Ca^2+^-dependence of phosphorylation at Ser201 by PKG *in vitro*, resulting in enhanced phosphorylation at 0.1 mM CaCl_2_, well into the physiological range of intracellular calcium concentration in rod photoreceptors^57^. Therefore, the increased susceptibility of bG161R/GCAP2 to phosphorylation at Ser201 *in vivo* is likely the cause of bG161R/GCAP2 retention at the inner segment, as it would trigger the binding of 14-3-3.

We have previously demonstrated that GCAP2 accumulation at this compartment in an unstable conformation results in severe toxicity. Therefore we propose that the mislocalization of the mutant protein contributes to the pathophysiology of hG157R/GCAP2 bearing patients. Future studies will be addressed at investigating the molecular basis of Ca^2+^-free GCAP2 toxicity at the inner segment compartment.

In this study we have also analyzed the effect of P50L/GCAP1 mutation on subcellular distribution. Unlike most GCAP1 blindness-associated mutations, P50L does not affect the Ca^2+^ sensitivity of cyclase regulation *in vitro*^19^. We reasoned that the P50L mutation could affect subcellular localization. However P50L/GCAP1 in rods showed a normal subcellular distribution, discarding this possibility.

Taken together, our results point to the RetGC/GCAPs complex being assembled at the inner segment, and transported to the rod outer segment as a protein complex. This initial study sets the ground to investigate the *in vivo* mechanisms regulating the assembly and transport of the RetGC/GCAPs, a central protein complex in photoreceptor physiology and the basis of many inherited retinal disorders.

## Materials and Methods

Pertaining to animal research, this study was conducted in accordance with the ARVO statement for the use of animals in ophthalmic and vision research and in compliance with acts 5/1995 and 214/1997 for the welfare of experimental animals of the autonomous community (Generalitat) of Catalonia, and approved by the ethics committee on animal experiments of the University of Barcelona. Transient transgenic mice for this work were obtained by transfecting recombinant DNAs on the GCAP1/GCAP2 double knockout mice that have been previously described^2^. This mouse strain lacks the expression of both GCAP1 and GCAP2.

### Generation of mammalian expression vectors for *in vivo* DNA electroporation

For generation of a plasmid DNA expressing hGCAP1 under the mouse opsin promoter (MOP), hGCAP1 cDNA was amplified from pET21b-hGCAP1 plasmid (a gift from Dr. James Hurley, University of Washington) with primers P1-P2 (Table 1) and inserted into the XhoI/BamHI sites of pMOP. The plasmid DNA for bGCAP2 expression has been previously described^2^. The array of GCAP1 and GCAP2 mutant constructs was obtained by site-directed mutagenesis (Quick-Change kit, Stratagene, San Diego, USA) and verified by sequencing. A plasmid encoding enhanced green fluorescent protein (EGFP) was injected with each expression vector of interest, to identify the area of injection [pL-UG plasmid, encoding EGFP under the ubiquitious C promoter, #L01GLUG001XA. Signaling Gateway, UCSD, USA].

**Table 1:** List of primers used for the generation of expression vectors.

Primers	Construct	Primer Sense	Primer Antisense
1–2	GCAP1-XhoI-BamHI	gcatgcctcgagatgggcaacgtgatggagggaaagtcagtg	gcatgcggatcctcagccggctgcctcagcggcctcgtcagc
3–4	GCAP1-G2A	cctccatcacgttggccatctcgaggctg	cagcctcgagatggccaacgtgatggagg
5–6	GCAP1-K23D	gagtgccaccagtggtacgacaagttcatgactgagtgc	gcactcagtcatgaacttgtcgtaccactggtggcactc
7–8	GCAP1-P50L	aagaacctgagcctgtcggccagccag	ctggctggccgacaggctcaggttctt
9–10	GCAP1-W94A	gtggaacagaagctccgcgcgtacttcaagctctatga	tcatagagcttgaagtacgcgcggagcttctgttccac
11–12	GCAP2-G2A	gggccaggatggcgcagcagttcag	ctgaactgctgcgccatcctggccc
13–14	GCAP2-G161R	ccttctggtggatgaaaatcgagatggtcagctg	cagctgaccatctcgattttcatccaccagaagg
15–16	GCAP2-S201D	gatctctcagcagaggcggaaagatgccatgttctgag	ctcagaacatggcatctttccgcctctgctgagagatc
17–18	GCAP2-S201G	ctcagcagaggcggaaaggtgccatgttc	gaacatggcacctttccgcctctgctgag

Primers used for the generation of GCAP1 and GCAP2 expression vectors. Primers were used in the cloning work or PCR-mediated site-directed mutagenesis, as indicated in Methods.

### *In Vivo* DNA electroporation in the retina

*In vivo* DNA electroporation in the retina following DNA injection in the subretinal space was performed as originally described^64^ with minor modifications. Plasmids were amplified in the *E. coli* DH10β and purified (Qiagen plasmid maxi kit, Germany). The DNA construct of interest (circular form) was mixed with pL_UG (expressing GFP) at a 2:1 molar ratio in PBS with 0,1% fast green dye, at a final concentration of 6 µg/µl. GCAPs-/- neonatal mice were anesthetized on ice, and the eyelid and sclera were punctured with a 30-gauge needle. For DNA injection, we used customized capillary glass pipettes [(#300048. Harvard Apparatus, Holliston, Massachusetts, USA) pulled in a Puller P-97 from Sutter Instruments according to the following parameters: heat = 650, Pull = 60, Velocity = 60, Time = 200]. The capillary glass pipettes were attached to a nanoinjector (Nanoject, Drummond Scientific, Broomall, PA, USA) and managed by a micromanipulator. After inserting the glass pipette in the eye, and carefully micromanipulating it to reach the subretinal space, 0,5-1 µl of the DNA solution was delivered. Tweezer-type electrodes were placed to softly hold the pup heads with the positive electrode over the injected eye, and five square pulses of 80 V of 50-ms duration with 950-ms intervals were applied (CUY21, Nepagene, Ichikawa, Chiba, Japan). Pups were left to recover, returned to their cage and raised normally. Mice were processed for analysis at postnatal day 25–30.

### Indirect Immunofluorescence detection assays of GCAP1 and GCAP2

For immunofluorescence localization assays, mice were sacrificed and eyes enucleated. Eyes were punctured with a needle and submerged in fixative (4% paraformaldehyde; 0.02% glutaraldehyde in phosphate buffer saline at pH7.4). The cornea was excised, and at 1 h in fixative, the lens was removed and the eyecups further fixed for a total of 2 h at room temperature. Eyecups were washed in PBS and infiltrated in acrylamide (8.4% acrylamide, 0.014% bisacrylamide in PBS pH7.4) for 14 h before acrylamide polymerization was induced, included in OCT compound and frozen in liquid nitrogen. Cryosections along the vertical axis of the eyecup were obtained at 20 µm-thickness using a CM1510S Leica cryostat (Leica Microsystems, Wetzlar, Germany).

A brief antigen retrieval protocol was applied, by incubating retinal sections with proteinase K in PBS (0.05 mg/ml) for 2 min, and heating at 70 °C for 10 seconds. Sections were washed and incubated in blocking solution (3% normal goat serum, 1% BSA, 0,1% Triton-X100 in PBS pH7.4) for 1 h at room temperature; first antibody in blocking solution without Triton-X100 (16 h at 4 °C); secondary antibody (1.5 h at room temperature), and fixed for 15 min in 4% paraformaldehyde prior to being mounted with Mowiol [Calbiochem #475904, San Diego, CA, USA]. Primary antibodies were the anti-GCAP1 pAb or anti-GCAP2 pAb generated in rabbit against whole recombinant proteins that have been previously described^2^. Both antibodies have been shown to be highly specific in GCAP1 and GCAP2 immunolocalization studies on murine retinas^39,41^. Rhodopsin antibody was mAb 1D4. Secondary antibodies were anti-Rabbit Alexa Fluor 555 (A-31572) and anti-Mouse Alexa Fluor 647 (A-21236), both from LifeTechnologies (Carlsbad, CA, USA).

Images were acquired at a laser scanning confocal microscope (Leica TCS-SL). Images were processed with Leica confocal software Lite and ImageJ. Colors were artificially assigned: green color was assigned to the 555-channel, and red color was assigned to the 647-channel. Regions of interest (ROI) were selected to quantify the green fluorescence either at the outer segment or the inner segment of each cell, and the percentage of GCAP protein at the outer segment was determined as: signal at the outer segment/(signal at the outer segment + signal at the inner segment).

Statistical Test. An unpaired two-way T-test was used to determine significant statistical difference of mutants versus WT using the GraphPad Prism6 software.

### bGCAP2 and bG161R/GCAP2 recombinant protein expression and *in vitro* phosphorylation assays

The plasmid for bGCAP2 bacterial expression consisted of bGCAP2 cDNA inserted into the NdeI-BamHI sites of pET15b (incorporates a His.tag at the NH2-terminus, Novagene, Merck Millipore) and has been previously described^41^. bG161R/GCAP2 expression vector was obtained by site-directed mutagenesis (Quick-Change kit, Stratagene, San Diego, USA) and verified by sequencing. Protein expression was performed on E. coli BL21(DE3) by chemical transformation, growth of bacterial cultures to an OD600 of 0.5 and induction of expression with 1 mM IPTG for 4 h at 37 °C. For myristoylation of GCAP2, cells were co-transformed with pBB131 plasmid encoding N-myristoyl transferase (NMT) (a gift from Dr. J. Gordon, Washington University School of Medicine, Missouri, USA) and free myristic acid was added to 50 μg/ml. GCAP2 and G161R/GCAP2 were insoluble and were purified from inclusion bodies. Briefly, inclusion bodies were obtained by sequential steps of bacterial lysis and sedimentation, in lysis buffer (100 mM sodium phosphate pH8, 150 mM NaCl, 1 mM PMSF, 0.5 mg/ml lysozyme). Purified inclusion bodies were solubilized in solubilization buffer (6 M GuHCl, 0.1 M NaH2PO4, 10 mM Tris-HCl pH = 8, 150 mM NaCl, 20 mM imidazole, 2.5 mM b-mercaptoetanol), clarified by centrifugation, filtered and loaded to pre-equilibrated 5 ml HisTrapTM Chelating HP Columns (GE Healthcare). Columns were extensively washed, and a protocol for on-column refolding was performed by exchanging 6 M urea for the 6 M guanidinium-HCl in running buffer (0.1 M NaH_2_PO_4_, 10 mM Tris-HCl, 150 mM NaCl, 20 mM imidazole, 2.5 mM b-mercaptoetanol, pH8.0) followed by a decreasing urea gradient (6 M to 0 M urea in running buffer, in 30 column volumes), using a Waters chromatography system. Proteins were eluted in 5 ml elution buffer (20 mM Hepes, 115 mM KCl, 10 mM NaCl, 500 mM imidazole, pH = 8), dialyzed against dilution buffer (20 mM Hepes, 115 mM KCl, 10 mM NaCl) until imidazole was <20 mM, and concentrated using 10 kDa MWCO Amicon Ultra-15 centrifugation filter units (Millipore). Protein stocks were kept at −80 °C until use. This GCAP2 purification procedure has been shown to yield active GCAP2 protein (e.g. recombinant GCAP2 protein used in GC assay in Fig. 3 of ref.^43^).

For *in vitro* phosphorylation of recombinant bGCAP2 and bG161R/GCAP2 by PKG, 20 μg of recombinant protein were incubated for 10 min in 40 μl of dilution buffer (20 mM Hepes, 115 mM KCl, 10 mM NaCl) with pre-fixed concentrations of EGTA or CaCl_2_: 5 mM EGTA; 10 mM CaCl_2_; 1 mM CaCl_2_ or 0.1 mM CaCl_2_. *In vitro* phosphorylation reactions were subsequently performed by addition of 5 μl of 10x phosphorylation reaction buffer (300 mM Tris-HCl pH 7.5; 5 mM MgCl_2_; 50 mM sodium phosphate pH 7.5; 60 mM DTT, 500 µM cGMP and 100 µM ATP), 3µCi of ^32^P-γATP (Perkin Elmer, Massachusetts, USA) and 5 units of PKGIα (Calbiochem, Billerica, MA) to a final volume of 50 μl. Reactions were incubated at room temperature for 30 min, and stopped by addition of Laemmli buffer. Samples were resolved by 15% SDS-PAGE. Following transfer to a nitrocellulose membrane, an autoradiograph of the ^32^P phosphorylation products was obtained by 24 h of exposure to a Kodak X-ray film. The nitrocellulose membrane was subsequently incubated with a pAb anti-GCAP2 and IRDye 800CW Goat Anti-rabbit IgG for GCAP2 immunodetection.

All data generated or analysed during this study are included in this published article (and its Supplementary Information files).

## Electronic supplementary material

Supplementary Material
