# Supplementary material for: Molecular determinants of Guanylate Cyclase Activating Protein subcellular distribution in photoreceptor cells of the retina
Source: Sci Rep. 2018 Feb 13;8:2903. doi: 10.1038/s41598-018-20893-1 (PMC5811540; doi:10.1038/s41598-018-20893-1)
Supplement: Supplementary file 1 — Supplementary Material [file 41598_2018_20893_MOESM1_ESM.pdf]

SUPPLEMENTARY MATERIAL

TITLE: Molecular determinants of Guanylate Cyclase Activating Protein subcellular distribution in photoreceptor cells of the retina.

Authors: Santiago López-Begines[1], Anna Plana-Bonamaisó[2], Ana Méndez[1,2*].

Affiliations: [1]Bellvitge Biomedical Research Institute (IDIBELL), Barcelona, Spain. [2]Department of Physiology, University of Barcelona School of Medicine-Bellvitge Health Science Campus, Barcelona, Spain.

Supplementary Figure S1. Subcellular distribution of GCAP1 in individual rod photoreceptor cells of GCAP1/2 knockout mice transfected with the wildtype GCAP1 construct. Cells that were taken into account for determination of GCAP1 distribution to rod outer segments are numbered. The cells presented in Fig 1 of the main text of the article are not included here, and hence the discontinued numbering. OS: outer segment; IS: inner segment.

WT/GCAP1

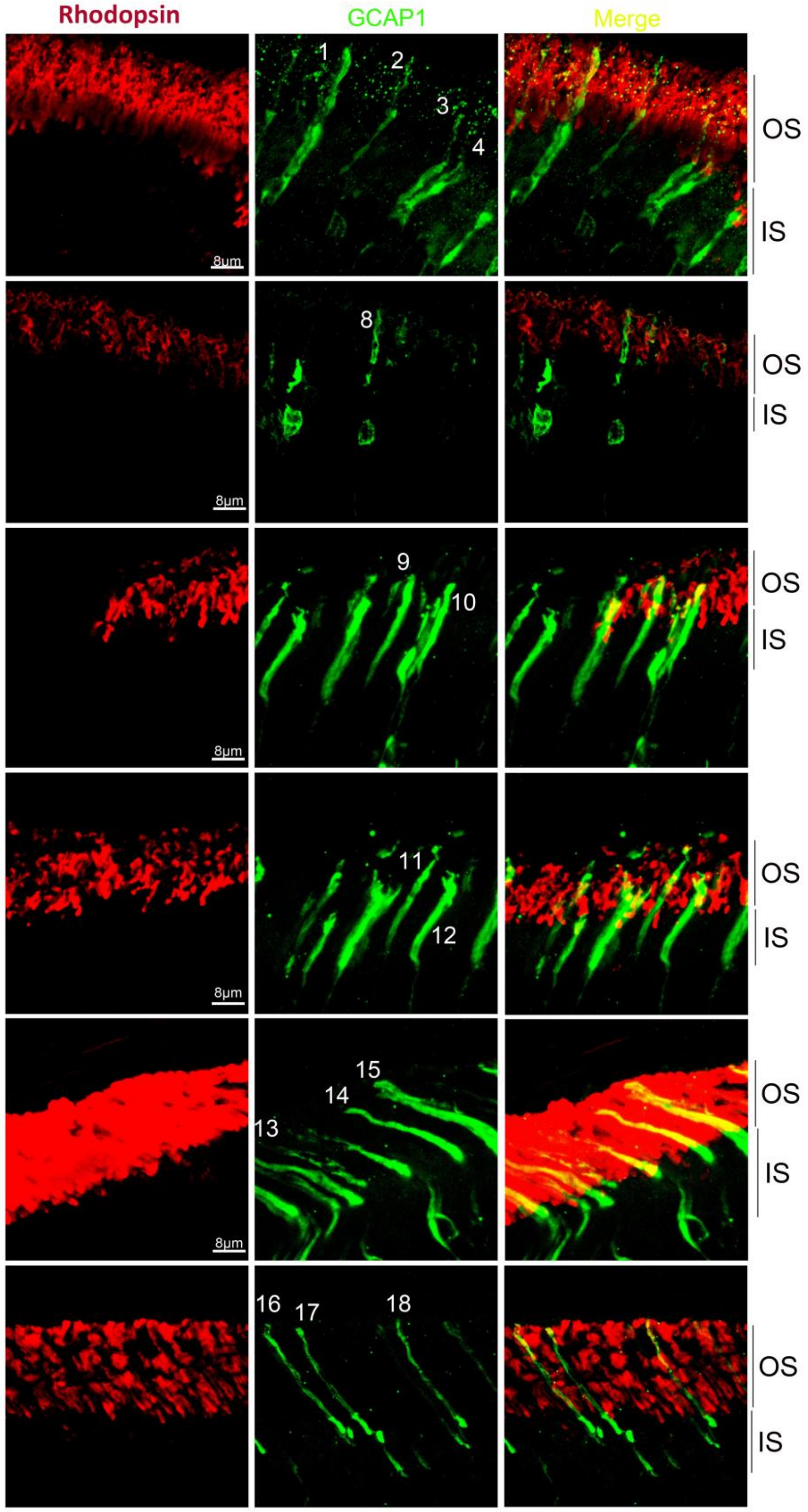

Rhodopsin
GCAP1
Merge
1
2
3
4
8
9
10
11
12
13
14
15
16
17
18
OS
IS
8μm

Supplementary Figure S2. Subcellular distribution of K23D/GCAP1 in individual rod photoreceptor cells of GCAP1/2 knockout mice transfected with the K23D/GCAP1 construct. The cells that were taken into account for determination of GCAP1 distribution to rod outer segments are numbered. The cells presented in Fig 1 of the main text of the article are not included here, and hence the discontinued numbering. OS: outer segment; IS: inner segment.

K23D/GCAP1

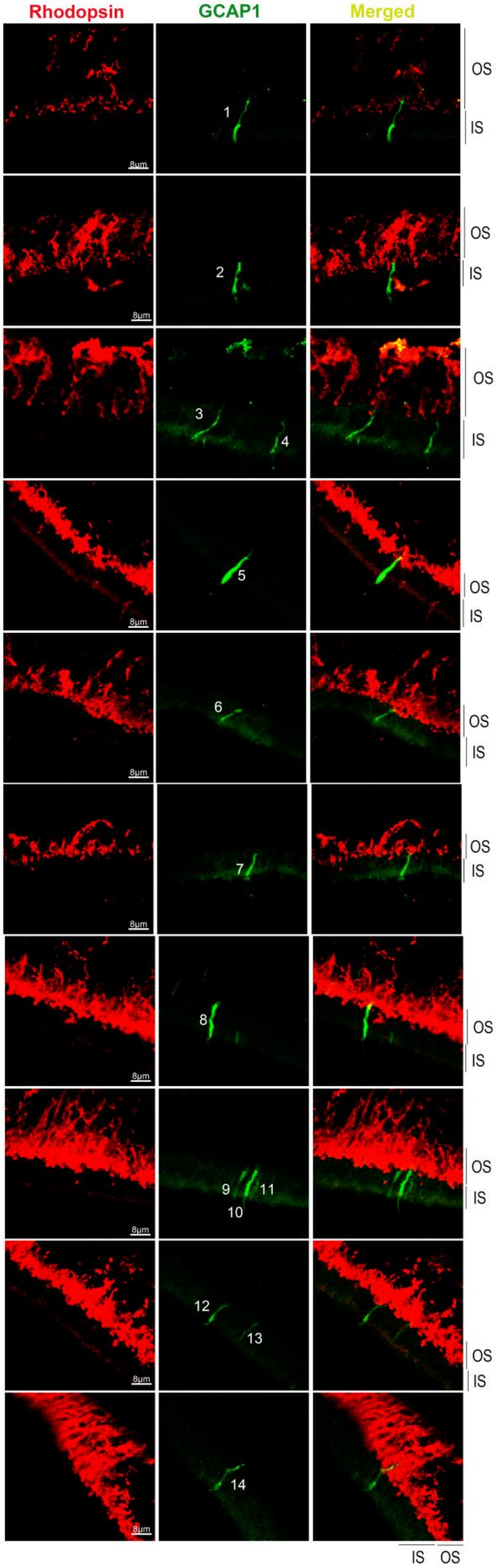

Supplementary Figure S3. Subcellular distribution of W94A/GCAP1 in individual rod photoreceptor cells of GCAP1/2 knockout mice transfected with the W94A/GCAP1 construct. Cells that were taken into account for determination of GCAP1 distribution to rod outer segments are numbered. The cells presented in Fig 1 of the main text of the article are not included here, and hence the discontinued numbering. OS: outer segment; IS: inner segment.

W94A/GCAP1

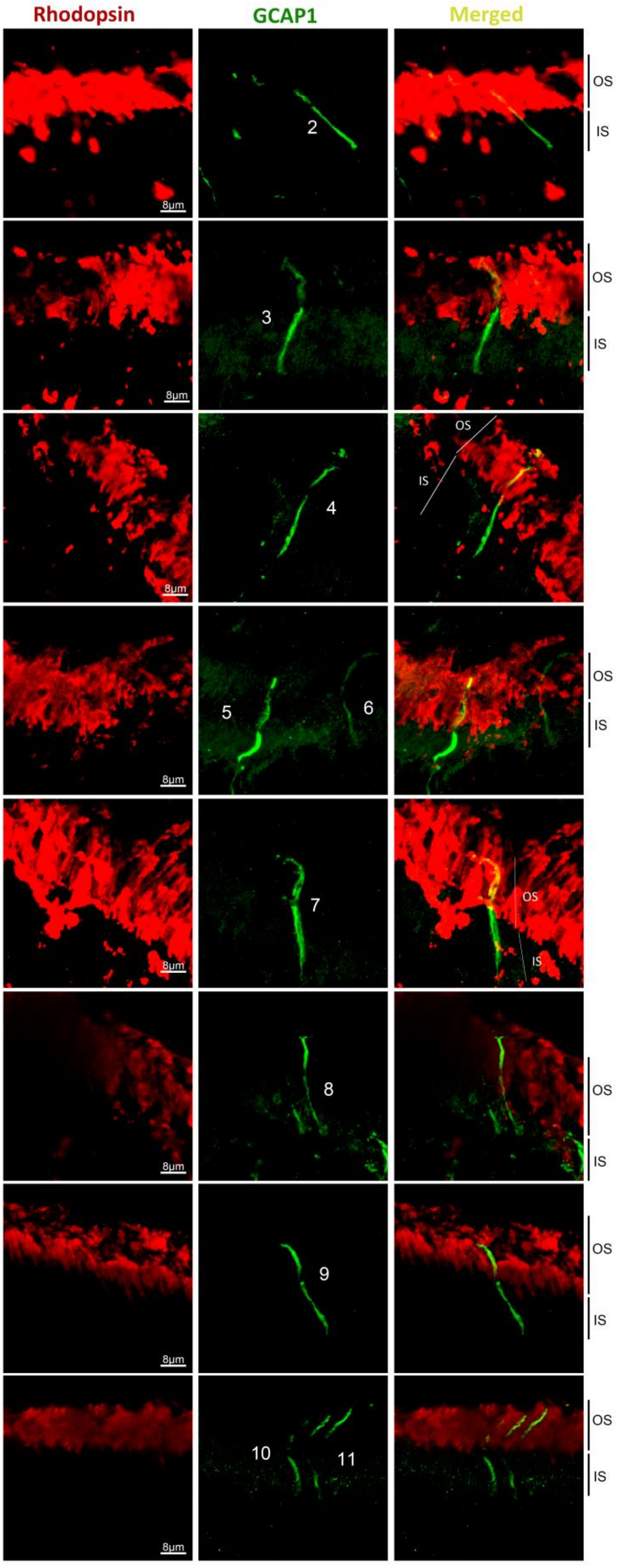

Supplementary Figure S4. Subcellular distribution of G2A/GCAP1 in individual rod photoreceptor cells of GCAP1/2 knockout mice transfected with the G2A/GCAP1 construct. The cells that were taken into account for determination of GCAP1 distribution to rod outer segments are numbered. The cells presented in Fig 1 of the main text of the article are not included here, and hence the discontinued numbering. OS: outer segment; IS: inner segment.

G2A/GCAP1

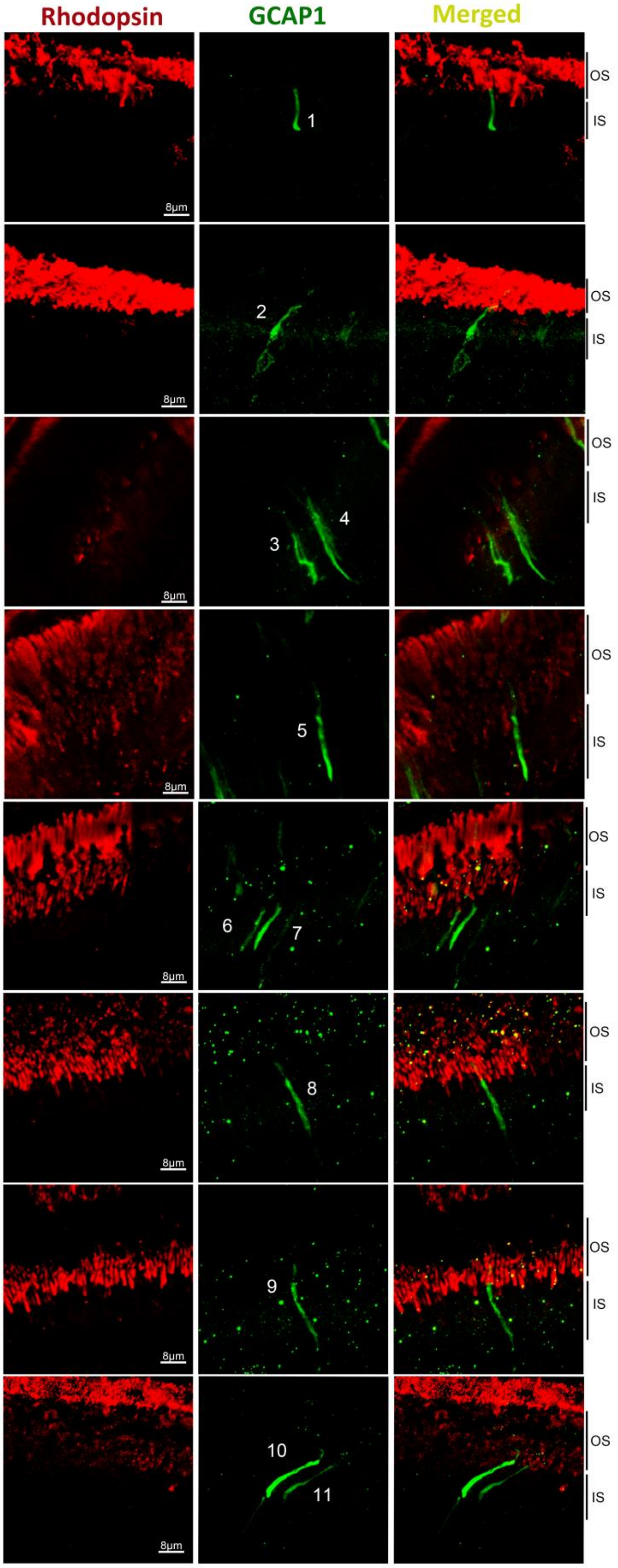

Supplementary Figure S5. Subcellular distribution of GCAP2 in individual rod photoreceptor cells of GCAP1/2 knockout mice transfected with wildtype GCAP2. Cells that were taken into account for determination of GCAP2 distribution to rod outer segments are numbered. The cells that are presented in Fig 2 of the main text of the article are not included here, and hence the discontinued numbering. OS: outer segment; IS: inner segment.

WT/GCAP2

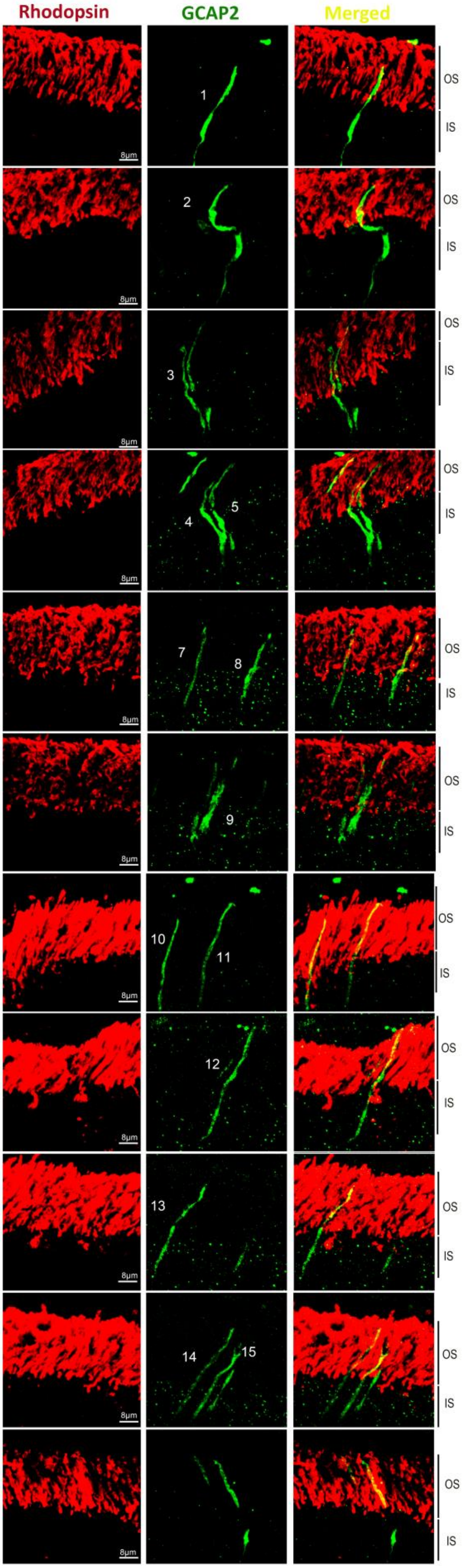

Supplementary Figure S6. Subcellular distribution of GCAP2 in individual rod photoreceptor cells of GCAP1/2 knockout mice transfected with constructs G2A/GCAP2; S201D/GCAP2; or S201G/GCAP2. Cells that were taken into account for determination of GCAP2 distribution to rod outer segments are numbered. The cells that are presented in Fig 2 of the main text of the article are not included here, and hence the discontinued numbering. OS: outer segment; IS: inner segment.

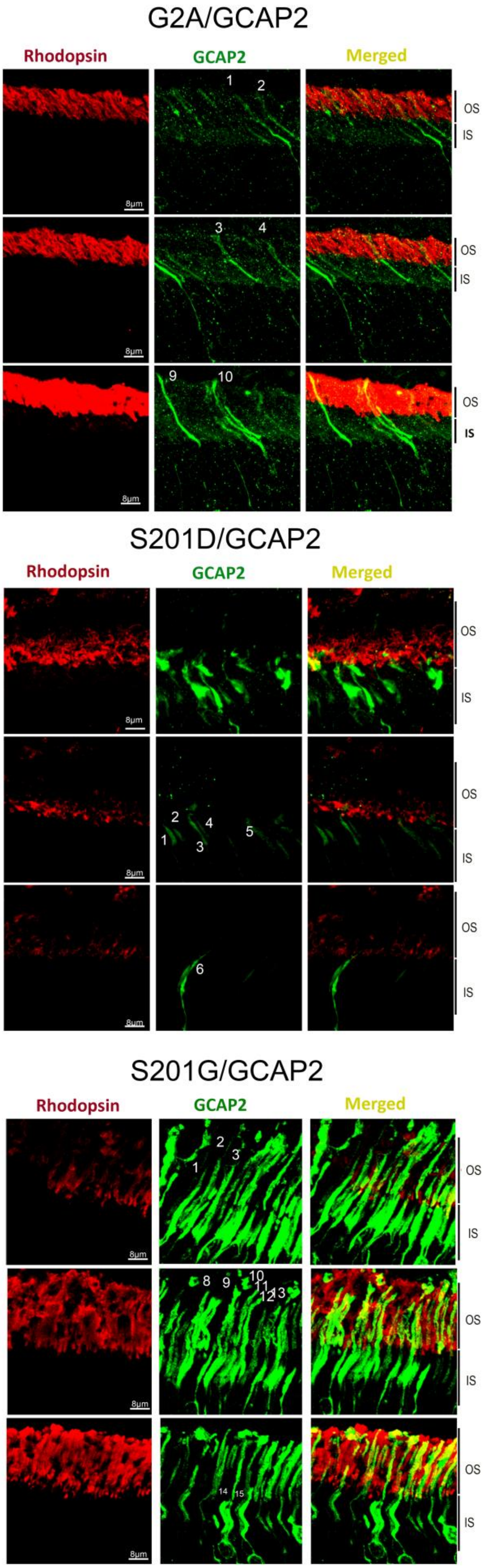
G2A/GCAP2
Rhodopsin
GCAP2
Merged
1
2
3
4
9
10
OS
IS
8µm
S201D/GCAP2
Rhodopsin
GCAP2
Merged
1
2
3
4
5
6
OS
IS
8µm
S201G/GCAP2
Rhodopsin
GCAP2
Merged
1
2
3
8
9
10
11
12
13
14
15
OS
IS
8µm

Supplementary Figure S7. Subcellular distribution of GCAP1 in individual rod photoreceptor cells of GCAP1/2 knockout mice transfected with P50L/GCAP1 construct. The cells that were taken into account for determination of GCAP1 distribution to rod outer segments are numbered. The cells that are presented in Fig 3 of the main text of the article are not included here, and hence the discontinued numbering. OS: outer segment; IS: inner segment.

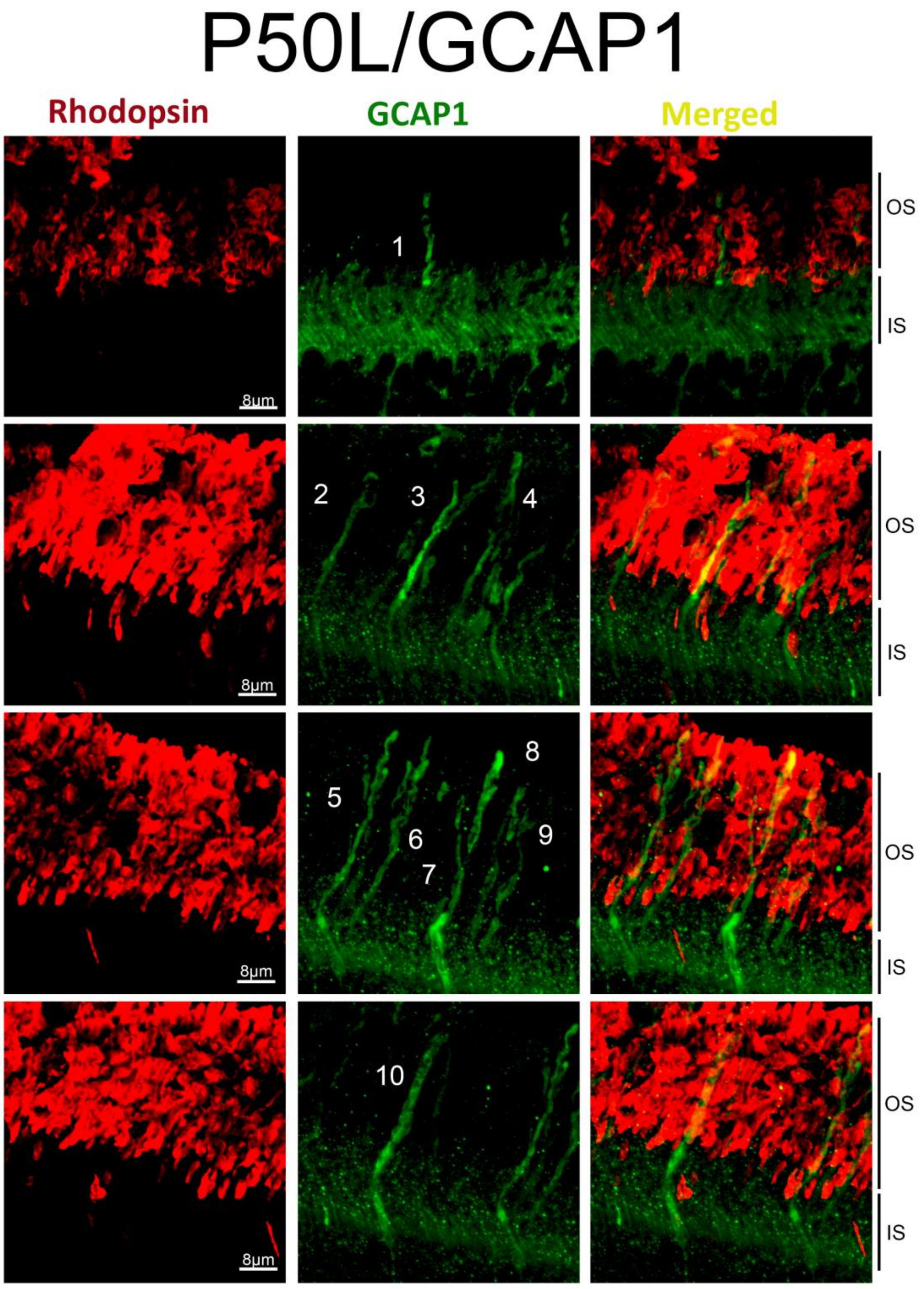
P50L/GCAP1
Rhodopsin
GCAP1
Merged
OS
IS
8µm
1
2
3
4
5
6
7
8
9
10

Supplementary Figure S8. Subcellular distribution of GCAP2 in individual rod photoreceptor cells of GCAP1/2 knockout mice transfected with bG161R/GCAP2. The cells that were taken into account for determination of GCAP2 distribution to rod outer segments are numbered. The cells that are presented in Fig 4 of the main text of the article are not included here, and hence the discontinued numbering. OS: outer segment; IS: inner segment.

G161R/GCAP2

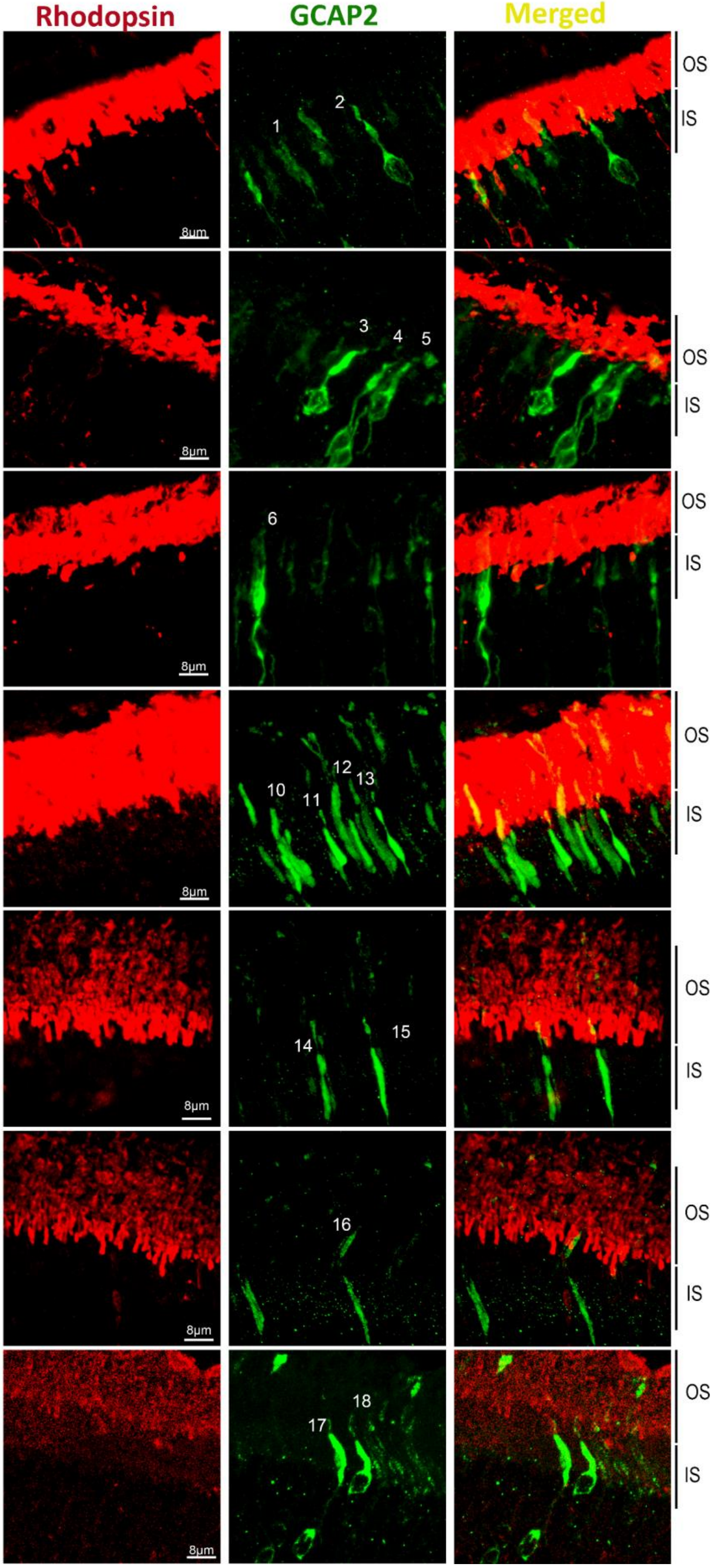
Rhodopsin
GCAP2
Merged
OS
IS
1
2
3
4
5
6
10
11
12
13
14
15
16
17
18
8μm
